# Impact of the COVID-19 pandemic on routine surveillance for adults with chronic hepatitis B virus (HBV) infection in the UK

**DOI:** 10.12688/wellcomeopenres.17522.2

**Published:** 2023-11-15

**Authors:** Cori Campbell, Tingyan Wang, David A. Smith, Oliver Freeman, Theresa Noble, Kinga A Várnai, Steve Harris, Hizni Salih, Gail Roadknight, Stephanie Little, Ben Glampson, Luca Mercuri, Dimitri Papadimitriou, Christopher R Jones, Vince Taylor, Afzal Chaudhry, Hang Phan, Florina Borca, Josune Olza, Frazer Warricker, Luis Romão, David Ramlakhan, Louise English, Paul Klenerman, Monique I. Andersson, Jane Collier, Eleni Nastouli, Salim I. Khakoo, William Gelson, Graham S. Cooke, Kerrie Woods, Jim Davies, Eleanor Barnes, Philippa C. Matthews

**Affiliations:** 1 Nuffield Department of Medicine, University of Oxford, Oxford, UK; 2 NIHR Oxford Biomedical Research Centre, University of Oxford, Oxford, UK; 3 NIHR Health Informatics Collaborative, Oxford University Hospitals NHS Foundation Trust, Oxford, UK; 4 Nuffield Department of Population Health, University of Oxford, Oxford, UK; 5 Department of Computer Science, University of Oxford, Oxford, UK; 6 NIHR Health Informatics Collaborative, Imperial College Healthcare NHS Trust, London, UK; 7 NIHR Imperial Biomedical Research Centre, London, UK; 8 Department of Infectious Disease, Imperial College London, London, UK; 9 Cambridge University Hospitals NHS Foundation Trust, Cambridge, UK; 10 NIHR Southampton Biomedical Research Centre, University Hospital Southampton NHS Foundation Trust, Southampton, UK; 11 University Hospital Southampton NHS Foundation Trust, Southampton, UK; 12 University College London Hospitals NHS Foundation Trust, London, UK; 13 Department of Infectious Diseases and Microbiology, Oxford University Hospitals NHS Foundation Trust, Oxford, UK; 14 Nuffield Department of Clinical Laboratory Sciences, University of Oxford, Oxford, UK; 15 Department of Hepatology, John Radcliffe Hospital, Oxford, UK; 16 Department of Clinical Virology, University College London Hospitals NHS Trust, London, UK; 17 School of Clinical and Experimental Sciences, Faculty of Medicine, University of Southampton, Southampton, UK; 18 Cambridge Liver Unit, Cambridge University Hospitals NHS Foundation Trust, Cambridge, UK; 19 Faculty of Medicine, Department of Infectious Disease, Imperial College London, London, UK

**Keywords:** hepatitis B virus, HBV, epidemiology, virology, viral hepatitis, COVID-19

## Abstract

**Background:**

To determine the impact of the COVID-19 pandemic on the population with chronic Hepatitis B virus (HBV) infection under hospital follow-up in the UK, we quantified the coverage and frequency of measurements of biomarkers used for routine surveillance (alanine transferase [ALT] and HBV viral load).

**Methods:**

We used anonymized electronic health record data from the National Institute for Health Research (NIHR) Health Informatics Collaborative (HIC) pipeline representing five UK National Health Service (NHS) Trusts.

**Results:**

We report significant reductions in surveillance of both biomarkers during the pandemic compared to pre-COVID-19 years, both in terms of the proportion of patients who had ≥1 measurement annually, and the mean number of measurements per patient.

**Conclusions:**

These results demonstrate the real-time utility of HIC data in monitoring health-care provision, and support interventions to provide catch-up services to minimise the impact of the pandemic. Further investigation is required to determine whether these disruptions will be associated with increased rates of adverse chronic HBV outcomes.

## Abbreviations

**Table:** 

ALT	Alanine transferase
CHB	Chronic HBV
COVID-19	Coronavirus Disease 2019
GHSS	Global Health Sector Strategy
HBV	Hepatitis B virus
HCC	Hepatocellular carcinoma
HIC	Health Informatics Collaborative
NHS	National Health Service
NIHR	National Institute for Health Research
SARS-CoV-2	Severe acute respiratory syndrome coronavirus 2
VL	Viral load
WHO	World Health Organization

## Introduction

Mortality and morbidity associated with the Coronavirus Disease 2019 (COVID-19) pandemic can be directly attributed to severe acute respiratory syndrome coronavirus 2 (SARS-CoV-2), but also result from indirect impacts on other conditions.

In order to progress towards elimination targets for Hepatitis B virus (HBV) infection ^1^ there is an urgent need for improvement of surveillance and treatment coverage. This will necessitate enhanced screening, followed by surveillance of individuals with chronic HBV (CHB) to determine treatment eligibility. Clinical follow-up includes routine monitoring of liver enzymes (e.g., alanine transferase (ALT)), hepatitis B virus (HBV) DNA viral load (VL), elastography, ultrasound, and occasionally liver biopsy ^2– 4^. In those receiving antiviral treatment, laboratory parameters are monitored to ensure virologic suppression is achieved and maintained, and to identify complications.

Surveillance using VL and liver enzymes is recommended, typically at intervals of 3–12 months, and more frequently among males at older age, those with biochemically active hepatitis, risk factors for hepatocellular carcinoma (HCC), complications of liver disease, recent diagnosis, or changing treatment plans ^5, 6^. COVID-19-attributable disruptions to HBV elimination efforts have been broadly described ^7, 8^, but we set out to quantify the specific impact on routine HBV surveillance in secondary care services in England using individual-level patient data.

## Methods

We undertook longitudinal and cross-sectional analyses using routinely-collected individual-level secondary care data collected across five NHS Trusts in England by the National Institute for Health Research (NIHR) Health Informatics Collaborative (HIC), as previously described ^9, 10^. HBV treatment data were available from three NHS Trusts. We investigated how ALT and VL surveillance varied between pre-COVID-19 (years 2016–2019) to COVID-19 (year 2020 and part of 2021), comparing surveillance metrics in patients on and off antiviral therapy.

We calculated cumulative probabilities of undergoing ≥1 ALT and HBV DNA VL measurement each year using the Kaplan-Meier method, comparing probabilities across years using the log-rank test. We quantified the mean number of ALT and HBV DNA measurements per 100 patients, during pre-COVID-19 and COVID-19 years, with 95% CIs calculated using the normal distribution (whereby standard error (SE) is estimated by $\frac{s}{\sqrt{n}}$ with *n* denoting sample size and *s* denoting standard deviation). Official UK government COVID-19 incidence data are presented ^11^. 

## Ethics approval

The research database for the NIHR HIC viral hepatitis theme was approved by South Central - Oxford C Research Ethics Committee (REF Number: 21/SC/0060). All methods in this study were carried out in accordance with relevant guidelines and regulations. The requirement for written informed consent was waived by South Central - Oxford C Research Ethics Committee, because data has been anonymised before its use and the study is retrospective.

## Results

### Coverage of ALT and VL testing (proportion tested at least once a year)

**Figure 1.**
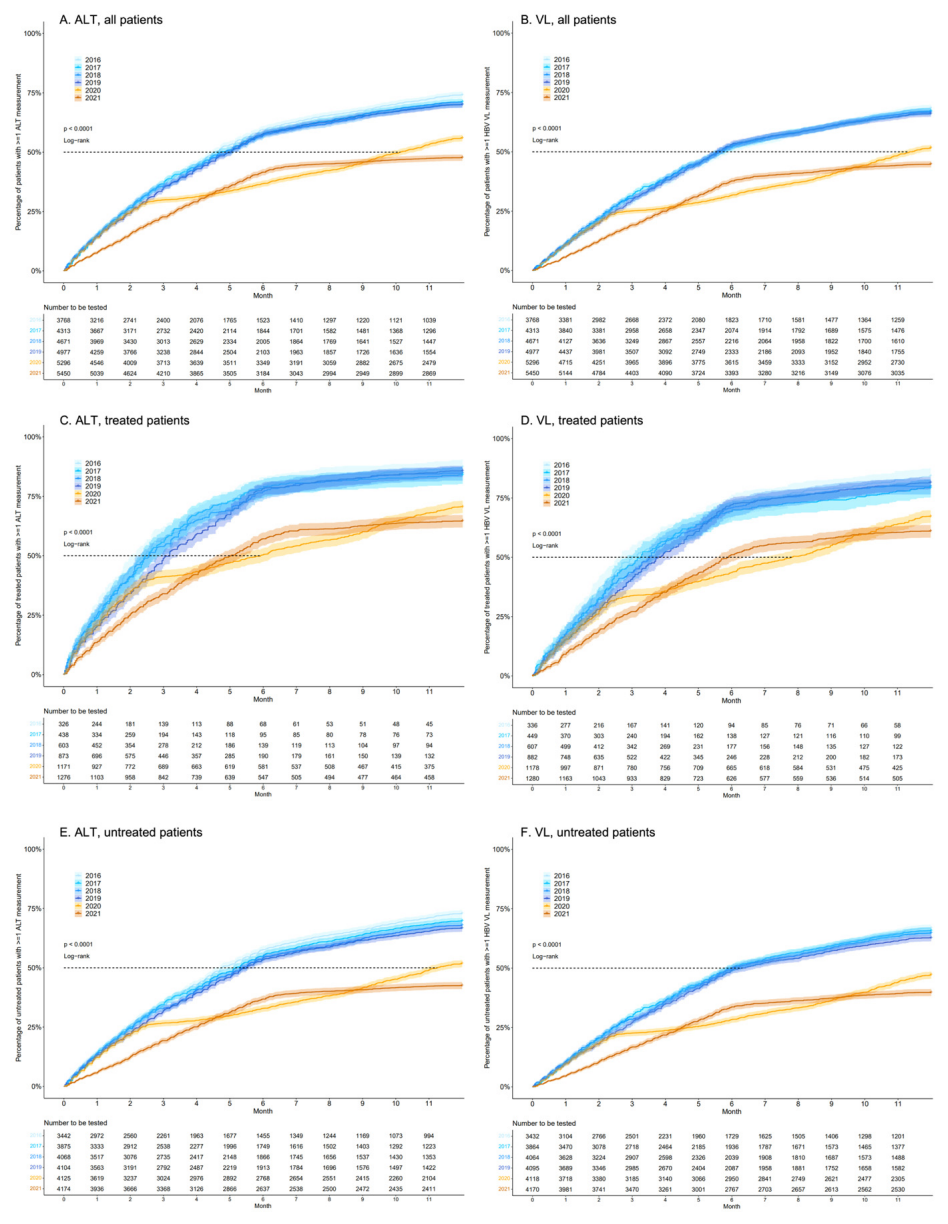
Kaplan-Meier plots demonstrating the cumulative proportion of adults with chronic Hepatitis B virus (HBV) infection undergoing routine laboratory surveillance. Plots show patients who have had ≥1 alanine transferase (ALT) (panels **A**, **C**, **E**) and ≥1 HBV DNA viral load (VL) (panels **B**, **D**, **F**) measurement each year for pre-COVID-19 (2016–19) and COVID-19 (2020 and part of 2021) years. For both ALT and VL, plots are stratified by treated (panels **C** and **D**, respectively) and untreated (panels **E** and **F**, respectively) patients. Dashed lines depict median time for 50% of the cohort to have the laboratory assessment undertaken. Cumulative probabilities for each year were calculated using the Kaplan-Meier method, comparing probabilities across years using the log-rank test, with 2016 serving as the reference/baseline year.

**Figure 2.**
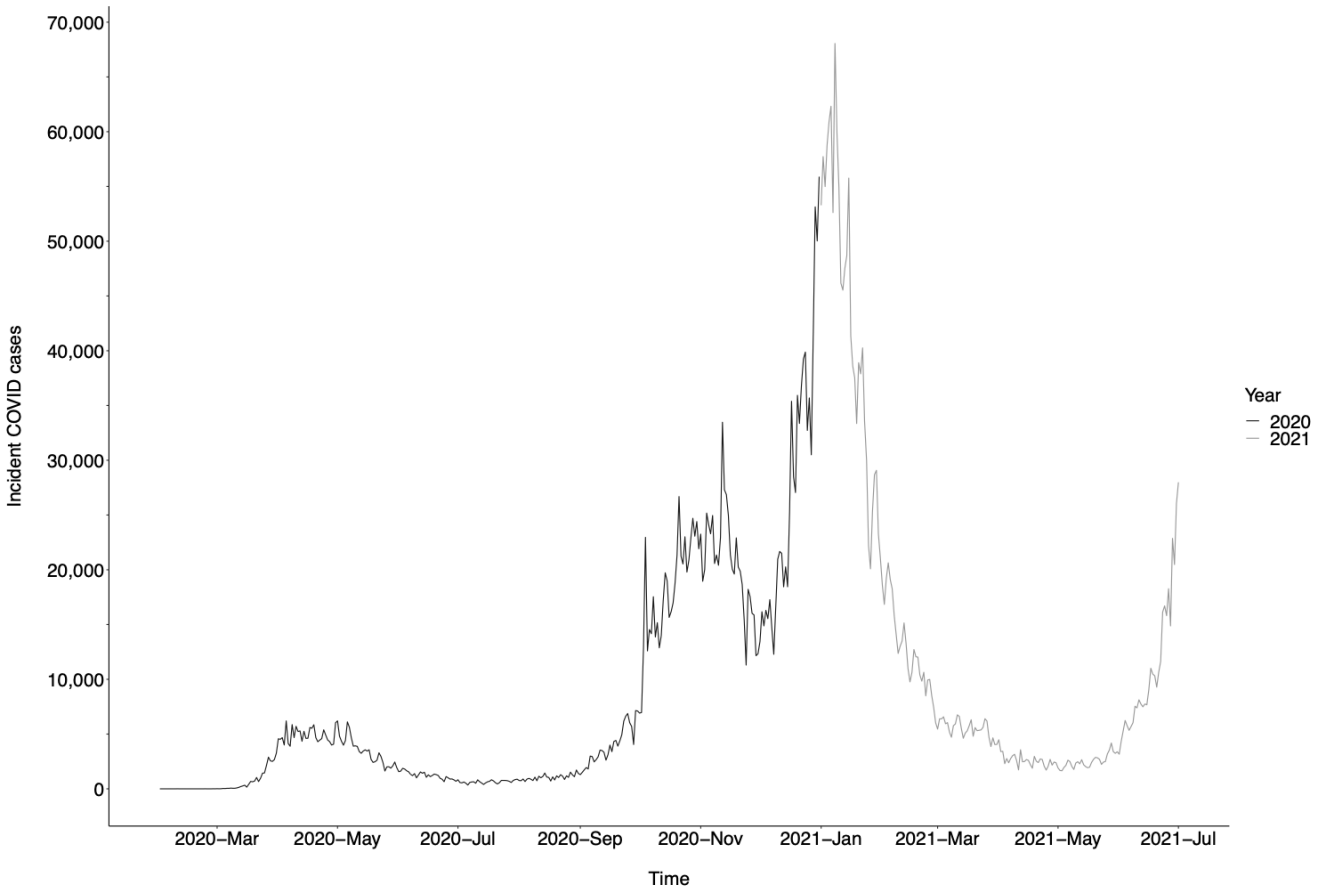
UK coronavirus disease 2019 (COVID-19) incidence, displayed as number of incident daily COVID-19 cases ^12^. Data from March 2020 to July 2021 are displayed.

Data were available from 3768 patients for the year 2016; this increased to 5450 by the year 2021. In 2016, 74.1% (95% CI 72.7-75.5%) of individuals with CHB had ALT measured on ≥1 occasion throughout the year. This significantly decreased to 56.0% (95% CI 54.6-57.3%) in 2020 and 47.7% (95% CI 46.4-49.0%) in 2021, suggesting a negative impact of the COVID-19 pandemic on routine clinical? surveillance ( Figure 1A). Similarly, throughout 2016, 68.4% (95% CI 66.9-69.9%) of patients had VL measured at least once, whilst this significantly decreased to 51.6% (95% CI 50.3-53.0%) in 2020 and 44.7% (95% CI 43.4-46.0%) in 2021 ( Figure 1B). From 2016 to 2019, median time to ALT/VL surveillance ranged between 138 and 175 days (out of 365 total in one calendar year). Median time to surveillance significantly differed in 2020, with durations of 308 and 345 days in 2020 for ALT and VL, respectively (Log-rank *P* < 0.0001). In 2021, 47.7% and 44.7% of individuals achieved ALT and VL surveillance by the end of the calendar year, respectively. COVID-19 incidence data are displayed in Figure 2.

### Coverage of ALT and VL testing stratified by treatment status

A subset of patients had treatment data available for years 2016 to 2021. Among treated patients, the proportion with ≥1 ALT measurement decreased significantly from 87.4% (95% CI 83.8-91.0%) in 2016 to 70.6% (95% CI 68.0-73.2%) in 2020, and 64.6% (95% CI 62.0-67.2%) in 2021 ( Figure 1C). Untreated patients demonstrated a reduction from 72.9% (95% CI 71.4-74.4%) in 2016 to 51.8% (95% CI 50.3-53.3%) in 2020 and 42.5% (95% CI 41.0-44.0%) in 2021 ( Figure 1E). In treated patients, median time to ALT surveillance ranged between 74 and 97 days from 2016-2019, and increased to 180 and 153 days in 2020 and 2021, respectively (Log-rank *P* < 0.0001). Differences in median time to surveillance were also observed in untreated patients, with median times to ALT surveillance ranging from 145 to 167 days during pre-COVID years and reaching 342 days in 2020 and with <50% individuals undergoing surveillance in 2021.

The proportion of treated patients with VL measured decreased from 84.2% (95% CI 80.3-88.1%) in 2016 to 67.2% (95% CI 64.6-69.9%) in 2020 and 61.0% (95% CI 58.3-63.7%) in 2021 ( Figure 1D). A decrease was also observed in untreated patients from 66.8% (95% CI 65.2-68.4%) in 2016 to 47.2% (95% CI 45.7-48.7%) in 2020 and 39.7% (95% CI 38.3-41.2%) in 2021 ( Figure 1F). In both treated and untreated individuals, cumulative probabilities comparing time to ALT and HBV VL measurement significantly differed across calendar years (Log-rank *P* < 0.0001). Median time to VL surveillance in treated patients ranged between 91 and 115 days from 2016-2019, increased to 238 and 153 days in 2020 and accordingly reduced to 180 days in 2021 (Log-rank *P* < 0.0001). In untreated patients, median time to VL surveillance ranged from 183 to 192 days during pre-COVID years, whilst in both 2020 and 2021 <50% of individuals underwent VL surveillance (Log-rank *P* < 0.0001).

### Frequency of ALT and VL testing

**Figure 3.**
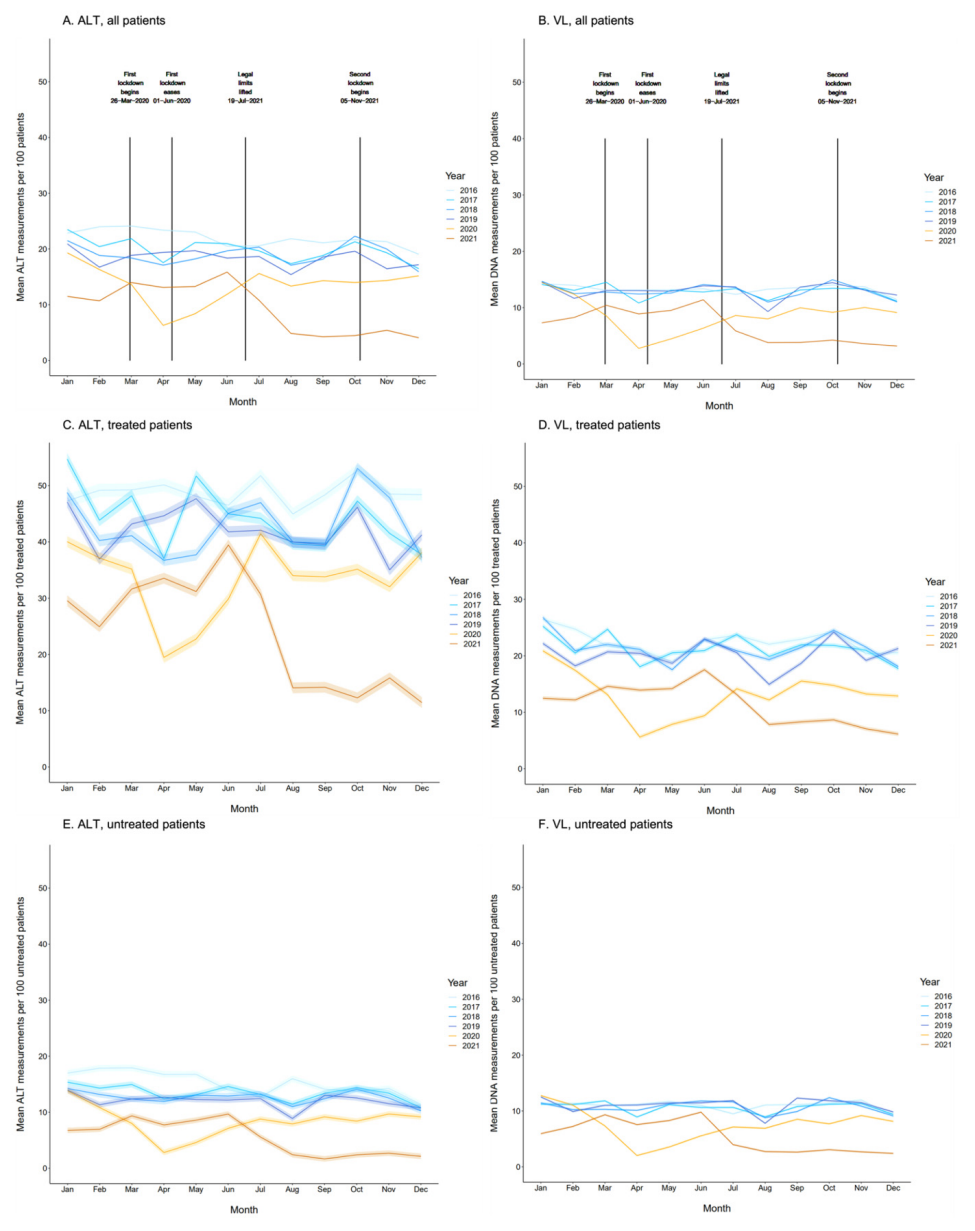
Mean numbers of ALT and HBV DNA VL measurements per 100 patients per month during pre-COVID-19coronavirus disease 2019 (COVID-19, 2016–19) and COVID-19 (2020 and part of 2021) periods. Data are shown overall and stratified by treatment status for both viral load (VL, panels **A**, **C**, **E**) and alanine transferase (ALT, panels **B**, **D**, **F**). Dates of UK national COVID-19 lockdowns are denoted in overall plots. 95% CIs were calculated using the normal distribution (whereby standard error (SE) is estimated by $\frac{s}{\sqrt{n}}$ with *n* denoting sample size and *s* denoting standard deviation).

Pre-COVID, the mean number of ALT measurements varied from 19 (95% CI 19-19) to 24 (95% CI 24-24) per 100 patients per month, with annual dips in August and December suggesting an impact of holiday periods when clinic activity may be reduced ( Figure 3A). Between March 2020 and December 2021, this range reduced to between 4 (95% CI 4-4) and 15 (95% CI 15-15), mean number of measurements per 100 patients per month remaining lower in these years as compared to pre-COVID years. The same trend was observed for VL ( Figure 3B), with mean numbers of measurements ranging from 9 (95% CI 9-9) to 15 (95% CI 15-15) per 100 patients pre-COVID, and from 3 (95% CI 3-3) to 11 (95% CI 11-12) during the COVID period, with nadirs of 3 measurements per 100 patients (95% CI 3-3) in April 2020 and December 2021.

### Frequency of testing by HBV treatment status

VL and ALT measurements were more frequent in treated ( Figure 3 C, D) compared to untreated ( Figure 3 E, F) patients throughout the study period. In treated patients pre-COVID, mean number of ALT measurements fluctuated between and within years but remained >35 measurements per 100 patients per month ( Figure 3C); hence, treated patients had ALT measured on average once every 4 months (in keeping with the routine interval for clinic visits in patients on treatment).

Mean numbers of ALT monthly tests fluctuated between 1 (95% CI 10-13) and 42 (95% CI 40-43) per 100 patients during the COVID-19 period but were generally significantly lower compared to pre-COVID years. This pattern was also observed for VL, with mean numbers of measurements ranging from 15 (95% CI 15-15) to 27 (95% CI 26-27) during pre-COVID years and 6 (95% CI 5-6) to 18 (95% CI 17-18) during the COVID period ( Figure 3D).

In untreated patients pre-COVID, mean number of ALT measurements per 100 patients per month ranged from 64 (95% CI 64-65) to 99 (95% CI 98-99), with a drop in the COVID period to between 23 (95% CI 22-23) and 74 (95% CI 73-74) ( Figure 3E). This pattern was also observed for VL, with pre-COVID and COVID measurements ranging from 8 (95% CI 8-8) to 12 (95% CI 12-13) and from 2 (95% CI 2-2) to 13 (95% CI 13-13) measurements per 100 patients, respectively ( Figure 3F).

## Discussion/conclusions

These data demonstrate the negative impact of the COVID-19 pandemic on routine clinical surveillance for patients with CHB infection in the UK. Reduction in rates of surveillance closely track SARS-CoV-2 incidence and thus periods of population lock-down.

During lockdown periods, much clinical care was switched to telemedicine ^13, 14^, intended to maintain contact between patients and healthcare providers, but at the detriment of laboratory monitoring. Although patients may have an option to attend phlebotomy appointments, services have been stretched, and clinicians and/or patients may have elected to defer routine blood tests. Telephone appointments are impractical for certain patients, and contact may have been lost altogether – for example, for those who do not communicate confidently in English. Furthermore, CHB patients who left the UK early in the course of the pandemic may have been unable to return due to travel restrictions, thereby foregoing routine surveillance. 

The impact of the pandemic was seen across treated and untreated individuals. However, the impact on ALT measurement was greater in those on treatment during lockdowns.

We were unable to investigate how imaging-based surveillance strategies were impacted due to lack of complete elastography and ultrasound data in our current dataset. Furthermore, as complications of CHB evolve over the long-term (months to years), it is not yet possible to quantify how service disruptions may impact incidence of HCC and other adverse endpoints. Surveillance disruption may be associated with an increased risk of CHB progression and delays in starting antiviral therapy. Further prospective data are required to determine the impact on long-term outcomes.

Our data represent only five large, urban centres, in the South East of England; trends may differ across settings and additional data will be required to determine the extent to which our observations apply in other regions. HIC data collection is being expanded ^10^, providing enhanced opportunities to incorporate a wider view of the UK over time. Effort and resources are required to refine telemedicine services and optimise access to laboratory surveillance ^15^, re-establish face-to-face services and catch-up on monitoring and interventions. Future analyses are warranted to investigate how imaging surveillance and telemedicine service uptake have been impacted when these data become available in the NIHR HIC database. Longitudinal follow-up is warranted to ascertain how COVID-attributable disruptions will impact the incidence of HCC and other relevant endpoints, and to ensure activity is re-aligned to support progress to elimination goals.

## Data Availability

Individual-level electronic health record were used to conduct this investigation. Data are anonymised and collected using National Institute for Health Research (NIHR) Health Informatics Collaborative (HIC) data resources. In order to ensure patient confidentiality and data privacy, raw underlying data cannot be made publicly available, and data are made available to researchers via a controlled access repository. The NIHR HIC Viral Hepatitis data repository is hosted by Oxford University Hospitals NHS Foundation Trust under a governance framework which includes a data sharing agreement and terms on confidentiality, contractual responsibilities, intellectual property, and publication. A scientific steering committee, comprised of at least one representative from each participating NIHR HIC site, meets regularly to review data collection, feedback progress on active projects, consider updates to the database, and review all data requests. Any potential collaborations are welcomed, and data are available to researchers on request following review by the steering committee. Further details are available at https://hic.nihr.ac.uk. Queries regarding data access should be directed to orh-tr.nihrhic@nhs.net.
