# Supplementary material for: Impact of the COVID-19 pandemic on routine surveillance for adults with chronic hepatitis B virus (HBV) infection in the UK
Source: Wellcome Open Res. 2023 Nov 15;7:51. Originally published 2022 Feb 11. [Version 2] doi: 10.12688/wellcomeopenres.17522.2 (PMC11077619; doi:10.12688/wellcomeopenres.17522.2)
Supplement: Peer review history

# Reviewer report: Reviewer response for version 2

**DOI:** 10.21956/wellcomeopenres.21838.r75872

**Published:** 2024-04-25

**Authors:** James Van Yperen

**Affiliations:** 1 University of Sussex, Brighton, England, UK

The authors present a study on the impact of the COVID-19 pandemic on routine surveillance for adults with a chronic hepatitis B virus infection in the UK. The authors use the Kaplan-Meier method (typically used for survival analysis) to analyse the cumulative probability of patients getting at least 1 test (either monitoring alanine transferase or virus DNA load) over the course of a year, generating estimates for 2016 up to 2021, and used a log-rank statistical test to compare these. Applying standard exploratory data analysis techniques, they provide statistics on the coverage of the two different tests, stratified by whether a patient has received treatment. The data used in the manuscript is not publicly available but can be made available by request to the steering committee (details available in the manuscript).

 The manuscript is well-written, but please thoroughly check for typos. The tests are appropriate, the results well represented and clear. Here are some scientific comments that could improve the manuscript:

1. Are surveillance levels going back to normal levels now? And if not, why do the authors think that is?
2. How could this drop in surveillance levels be mitigated in future pandemics causing lockdowns? For example, using home tests to check the viral load?
3. It would be interesting to see the distribution of the number of measurements per patient over the year. This could shed light on behaviour (e.g., lockdown caused a bimodal distribution with some not getting any measurements and some getting a lot).
4. I am not sure what Figure 2 is adding to the manuscript right now, other than a reminder on the COVID-19 cases?
5. The “methods” summary is not indicative of what methods you employed for your analysis, it should include the statistical methods and tests employed.

Is the work clearly and accurately presented and does it cite the current literature?

Yes

If applicable, is the statistical analysis and its interpretation appropriate?

Yes

Are all the source data underlying the results available to ensure full reproducibility?

Partly

Is the study design appropriate and is the work technically sound?

Yes

Are the conclusions drawn adequately supported by the results?

Yes

Are sufficient details of methods and analysis provided to allow replication by others?

Yes

Reviewer Expertise:

Mathematical modelling; mathematics of public health; operational research; nowcasting and forecasting using mathematics; systems modelling

I confirm that I have read this submission and believe that I have an appropriate level of expertise to confirm that it is of an acceptable scientific standard.

# Reviewer report: Reviewer response for version 1

**DOI:** 10.21956/wellcomeopenres.19374.r54846

**Published:** 2023-02-28

**Authors:** Dorcas Obiri-Yeboah

**Affiliations:** 1 Clinical Microbiology/Public Health Unit, Cape Coast Teaching Hospital, Cape Coast, Ghana; 2 Department of Microbiology and Immunology, School of Medical Sciences, University of Cape Coast, Cape Coast, Ghana

Thank you for the manuscript. This paper focused on how the pandemic impacted routine care for CHBV patients on treatment and those not on treatment. A number of articles have been published which looked at similar issues for other patients like HIV clients and other sin routine care prior to the pandemic. I am happy about the approach used here where the dataset set used is verifiable and the number of data points is large enough. The analysis has been done rigorously enough and clearly brings out the fact that laboratory services were very much negatively impacted compared with other aspects which telemedicine provided a solution.

 I am very happy and did not see any significant point to make to improve this manuscript. It is well written. The authors could propose or give some suggestions as to how this impact particularly on laboratory services could be mitigated in future similar situations. That would be helpful. And again what do you consider as further research that could offer more insight into what has been reported here? I am also wondering why the team did not look at the laboratory results where available and see if there was any trend pre- and during the pandemic? That could have implications for the care received.

Is the work clearly and accurately presented and does it cite the current literature?

Yes

If applicable, is the statistical analysis and its interpretation appropriate?

Yes

Are all the source data underlying the results available to ensure full reproducibility?

Yes

Is the study design appropriate and is the work technically sound?

Yes

Are the conclusions drawn adequately supported by the results?

Yes

Are sufficient details of methods and analysis provided to allow replication by others?

Yes

Reviewer Expertise:

Virology, infectious disease epidemiology, laboratory science

I confirm that I have read this submission and believe that I have an appropriate level of expertise to confirm that it is of an acceptable scientific standard.
